# Underwater indirect clipping for colonic diverticular bleeding

**DOI:** 10.1055/a-2836-1528

**Published:** 2026-04-15

**Authors:** Taiki Aoyama, Gentaro Sindo, Kenjiro Shigita, Naoki Asayama, Akira Fukumoto, Shinji Nagata

**Affiliations:** 1 13697 Department of Gastroenterology, Hiroshima City North Medical Center Asa Citizens Hospital, Hiroshima, Japan; 2 13697 Department of Endoscopy, Hiroshima City North Medical Center Asa Citizens Hospital, Hiroshima, Japan

Indirect clipping is a simple and widely used endoscopic therapy for colonic diverticular bleeding (CDB); however, it is associated with higher rebleeding rates than direct clipping or endoscopic band ligation (EBL 1 2 ). This limitation is attributed to the inability of indirect clipping to directly treat the exposed vessel at the dome of the diverticulum. In contrast, EBL achieves hemostasis by eradicating the entire diverticulum and inducing subsequent scarring 3 . Nevertheless, indirect clipping remains attractive because of its technical simplicity and favorable safety profile.

**Fig. 1:**
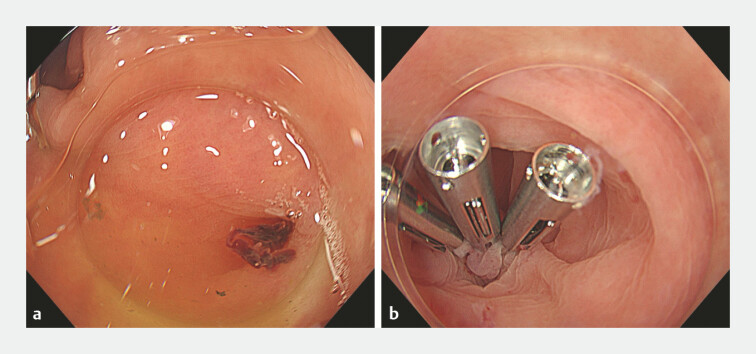
Identification and treatment of the culprit diverticulum. **a** Colonoscopy after bowel preparation identified a diverticulum with an adherent clot as the bleeding source. **b** Underwater indirect clipping achieved complete closure of the diverticulum.

**Fig. 2:**
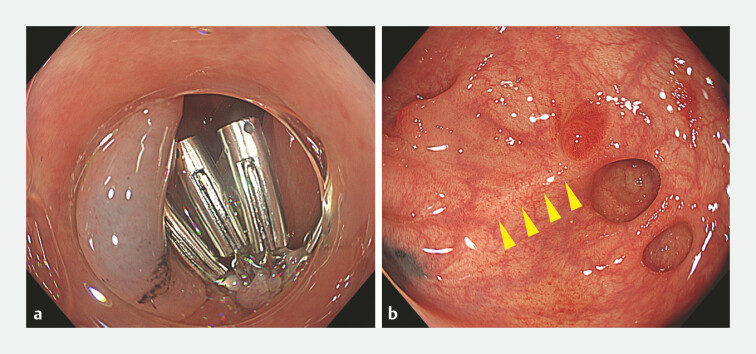
Post-treatment findings. **a** Tattooing was performed adjacent to the lesion after clipping. **b** Follow-up colonoscopy at 2 months showed scarring at the treatment site and disappearance of the responsible diverticulum.

A 73-year-old woman was admitted with hematochezia. Colonoscopy identified a diverticulum with an adherent clot as the bleeding source ( Fig. 1 **a** ). Because many diverticula were closely clustered, inversion and band ligation of the responsible diverticulum were considered technically difficult. In addition, the exposed vessel at the dome was unsuitable for direct clipping. Therefore, indirect clipping was selected ( Video 1 ). To achieve compression not only of the diverticular orifice but also of the dome, the colonic lumen was filled with water to minimize luminal distension 4 . The diverticulum was then closed by grasping and capturing a substantial amount of the surrounding mucosa using endoclips (SureClip; Micro-Tech, China), achieving complete closure ( Fig. 1 **b** ). In the underwater environment, the colonic wall became soft and pliable, allowing stable and secure tissue capture over a wider mucosal area 5 . Immediate hemostasis was achieved without adverse events. Tattooing was performed adjacent to the lesion ( Fig. 2 **a** ). The patient was discharged 4 days after treatment. Follow-up colonoscopy at 2 months showed scarring at the treatment site and disappearance of the responsible diverticulum, resembling the appearance after EBL ( Fig. 2 **b** ). No rebleeding occurred during the 6-month follow-up period.

Colonoscopy identified a diverticulum with an adherent clot as the bleeding source. Underwater indirect clipping achieved complete closure. Follow-up colonoscopy showed scarring and disappearance of the responsible diverticulum.Video 1

This case suggests that underwater indirect clipping is a simple, safe, and effective therapeutic option for CDB, particularly when direct clipping or EBL is technically challenging.

Endoscopy_UCTN_Code_TTT_1AQ_2AZ
